# Artificial intelligence-based integration of imaging, exposome, and multi-omics data for immune-related biomarker discovery and precision prevention in breast cancer

**DOI:** 10.3389/fimmu.2026.1870533

**Published:** 2026-09-14

**Authors:** Yuehong Xu, Hongbo Li

**Affiliations:** Department of Breast and Thyroid Surgery, Jinhua People’s Hospital, Jinhua, Zhejiang, China

**Keywords:** artificial intelligence, breast cancer, exposome (environmental exposure profiling), immune dysregulation, multi-omics integration, precision prevention

## Abstract

Breast cancer management still depends on early detection, risk assessment, and individualized treatment. Imaging is used throughout screening, diagnosis, treatment evaluation, and follow-up. For this reason, artificial intelligence (AI) has become an active area in breast cancer imaging, especially in mammography, ultrasound, MRI, and radiomics. These methods may help describe tumor phenotype, estimate clinical risk, and predict treatment response. In daily practice, however, similar imaging findings do not always mean the same biology. Patients with similar images may have different molecular features, immune status, exposure histories, and treatment outcomes. Imaging models alone are therefore not enough to explain the heterogeneity of breast cancer. Environmental and lifestyle exposures, metabolic status, and immune regulation may also influence tumor development and prognosis. This review discusses imaging AI as a practical starting point for biomarker discovery in breast cancer. It also considers how exposome and multi-omics data may improve the biological interpretation of imaging features, and how immune dysregulation may connect external exposure, molecular change, and imaging phenotype. Current barriers include fragmented data, limited external validation, weak interpretability, fairness concerns, and difficulty in clinical implementation. Future work should focus less on building larger models alone, and more on developing transparent and validated tools that clinicians can use for risk stratification, biopsy planning, treatment-response prediction, and precision prevention. This highlights the need to integrate imaging features with multi-omics data to better interpret tumor biology and immune status.

## Introduction

Breast cancer remains one of the most common malignancies among women worldwide, and its diagnosis, treatment, and follow-up impose a substantial burden on health-care systems and patients (1). Imaging has a central role in breast cancer management, from population-based screening and treatment monitoring to post-treatment surveillance (2). Previous studies have shown that machine learning systems can achieve high diagnostic performance in selected breast cancer screening tasks (3, 4). More recent work has expanded from lesion detection to radiomics, molecular subtype prediction, lymph node assessment, treatment response evaluation, and risk stratification (5, 6). These developments are consistent with the broader movement toward high-performance and deep learning-supported medicine (7, 8). This shift has a clear clinical background. The value of imaging is no longer limited to detecting a lesion; it also provides information for subsequent treatment decisions. When facing similar imaging findings, clinicians often need to determine whether a lesion requires biopsy, whether a patient is suitable for neoadjuvant therapy, whether follow-up intensity should be adjusted, and whether a tumor that appears localized may still have aggressive biological behavior. Imaging AI that can help answer these questions is closer to real clinical needs.

The heterogeneity of breast cancer results from the combined effects of genetic susceptibility, molecular alterations, environmental exposures, metabolic status, the tissue microenvironment, and immune regulation (9). Radiomics can capture tumor morphology, vascular distribution, texture, and spatial heterogeneity (10, 11). However, its explanatory power remains limited when imaging features are interpreted without biological context (12). Standardized feature extraction and reporting are also necessary before radiomics can be compared reliably across studies (13). The exposome (environmental exposure profiling) provides another perspective by focusing on the cumulative effects of environmental, lifestyle, occupational, chemical, nutritional, and social factors across the life course (14, 15). Recent exposome (environmental exposure profiling) frameworks further emphasize the need to connect environmental measurements with molecular biology and disease mechanisms (16, 17). In breast cancer, these exposures may influence risk and disease progression through endocrine disruption, chronic inflammation, oxidative stress, metabolic remodeling, and immune dysregulation (18, 19). Environmental chemical exposures are also supported by epidemiological evidence informed by biological mechanisms (20). For these reasons, a biomarker framework with greater clinical relevance should attempt to connect imaging phenotypes with exposome (environmental exposure profiling) and multi-omics data. Such a framework may help explain why similar imaging appearances are associated with different outcomes and may help identify patients whose tumors are related to exposure-associated inflammation, immune escape, or treatment resistance mechanisms. This review uses imaging AI as the starting point to discuss its role in breast cancer biomarker discovery, and further examines the potential value of exposome (environmental exposure profiling), multi-omics, and immune-related data in improving biomarker interpretability and clinical translation.

## Imaging AI as an entry point for biomarker discovery

Breast imaging is naturally suited to AI research because it is data-rich, relatively reproducible, and closely linked to clinical decision-making. Early applications mainly focused on screening and classification tasks, such as distinguishing benign from malignant lesions on mammography (21). These studies demonstrated technical feasibility, but systematic reviews also highlighted that high accuracy in selected datasets does not necessarily translate into better outcomes in real-world screening settings (4, 22). Therefore, clinical evaluation of AI diagnostic models requires attention to study design, external validation, calibration, and clinical effect (23). This concern is consistent with wider discussions on how artificial intelligence should be integrated into high-performance medicine rather than treated as a stand-alone technical result (7). Radiomics has extended the scope of imaging by converting medical images into quantitative data rather than relying only on visual impressions (11, 24). In breast cancer research, radiomic features have been used to analyze tumor subtype, axillary lymph node status, response to neoadjuvant therapy, prognosis, and recurrence risk (6, 25). However, radiomics also has clear limitations. Many imaging features remain descriptive: they may indicate risk but do not necessarily explain where the risk comes from (12). A model may identify a high-risk texture pattern, but clinicians still need to know whether that pattern reflects cellular density, necrosis, angiogenesis, immune infiltration, or differences introduced by scanning parameters and image processing. This distinction is important. A biomarker is more likely to be accepted clinically only when it helps explain disease behavior or guide treatment. Imaging risk scores without a biological basis may perform well in development cohorts, but they may be difficult to apply stably in external populations. Standardized image acquisition, feature extraction, and reporting are therefore essential for improving reproducibility and external validity (13, 23).

Recent studies in TNBC have demonstrated that cancer stem cell properties and metabolic reprogramming are closely linked to multi-omics signatures, including R-loop dynamics and lysine beta-hydroxybutyrylation (Kbhb) (26, 27). These findings highlight the importance of integrating imaging biomarkers with molecular and immune features to accurately capture tumor heterogeneity. Multi-omics integration provides a route for linking imaging biomarkers with disease biology. Genomic and transcriptomic data can help determine whether imaging phenotypes are associated with proliferation, hormone receptor signaling, HER2 activity, DNA repair deficiency, epithelial-mesenchymal transition, or immune-related pathways (28, 29). More general multi-omics approaches also provide useful frameworks for connecting disease phenotypes with molecular regulation across different biological layers (30). These methods require careful interpretation because data integration can increase both biological insight and statistical complexity (31). Proteomic and metabolomic data can further provide information on signaling activity, metabolic adaptation, oxidative stress, and treatment response (32). Microbiome-related studies also suggest potential effects on estrogen metabolism, systemic inflammation, and immune regulation (33).

From a clinical perspective, the most direct value of multi-omics integration is improved interpretation of results. If a specific imaging feature is associated with immune infiltration, inflammatory cytokine activity, or antigen presentation pathways, it is no longer only a statistical pattern, but may become a non-invasive surrogate of a biological process. This is particularly meaningful when tissue sampling is limited, repeated biopsy is difficult, or a single biopsy cannot fully reflect spatial heterogeneity. The immune microenvironment has received increasing attention in recent years. Tumor-infiltrating lymphocytes have prognostic and predictive value in several breast cancer subtypes (34, 35). The broader tumor immune microenvironment, including macrophages, cytokines, stromal cells, and immune checkpoints, also affects tumor progression and treatment response (36, 37). In triple-negative breast cancer, evolving systemic treatment strategies further highlight the importance of immune-related biomarkers (38). Immunotherapy and PD-L1 assessment provide examples of how immune markers may influence clinical decision-making (39, 40). Deep learning analysis of pathology images has also shown that spatial immune organization can be linked with molecular features (41). If imaging features can be cross-validated with spatial transcriptomic distribution, circulating immune markers, or tissue immune phenotypes, clinicians may interpret imaging findings more specifically and generate clearer hypotheses for subsequent validation. Nevertheless, multi-omics integration is not simply the stacking of data. Imaging, genomics, proteomics, metabolomics, microbiomics, and immunomics differ in acquisition methods, data scale, noise level, missingness, spatial resolution, and temporal dimension. Directly combining a large number of variables may instead lead to overfitting. More reliable approaches require standardized data collection, clearly defined clinical endpoints, biologically informed feature selection, external validation, and transparent reporting (29, 31).

## Exposome, immune dysregulation, and breast cancer risk

The exposome (environmental exposure profiling) expands breast cancer biomarker research beyond genetic risk and intrinsic tumor biology. Exposure factors related to breast cancer include reproductive and hormonal factors, obesity, diet, alcohol intake, physical activity, air pollution, endocrine-disrupting chemicals, circadian rhythm disruption, psychosocial stress, and residential environment (18, 20). The exposome (environmental exposure profiling) concept emphasizes that these exposures should be considered cumulatively rather than as isolated variables (15, 16). A public health-oriented exposome (environmental exposure profiling) paradigm also helps connect environmental context with long-term disease risk (17). Air pollution has been investigated as one measurable environmental exposure associated with breast cancer risk (19). These factors usually do not act alone, but interact with genetic susceptibility, metabolic status, breast tissue composition, and immune regulation. Immune dysregulation may be an important intermediate link between external exposures and breast cancer development and prognosis. Chronic inflammation is involved in the pathogenesis of many diseases across the life course and may also contribute to breast cancer progression and prognosis (42). Environmental and lifestyle factors can promote oxidative stress, metabolic abnormalities, microbiome alterations, and persistent inflammatory signaling. These changes further affect immune surveillance, cytokine networks, antigen presentation, and the balance between antitumor immunity and immune escape.

This perspective has practical relevance for precision prevention. Current breast cancer risk models mainly rely on demographic, reproductive, family history, genetic, and clinical variables (43). These models are valuable in clinical practice, but they may still have limited ability to capture modifiable exposures and immune-mediated risk. Adding exposome (environmental exposure profiling) data may help identify patients whose risk is influenced by metabolic, environmental, or social factors (15, 19). Further integration with immune and molecular markers may help distinguish exposure-related risk from risk that already reflects biological activity. The real difficulty lies in measurement. Exposome data are often incomplete and vary over time, across regions, and across social environments (16, 17). Indicators such as air pollution, occupational history, dietary patterns, physical activity, and neighborhood deprivation are usually measured with less precision than tumor sequencing or standardized imaging. AI may help integrate these incomplete data, but if study design does not adequately account for confounding factors and epidemiological bias, models may amplify erroneous signals and compromise the reliability of conclusions.

## An AI integration framework for clinical translation

In this context, the value of AI is not limited to processing large-scale data. More importantly, it may build connections among imaging, clinical variables, exposure information, and molecular features. Supervised learning models can combine imaging, clinical, and molecular variables to predict predefined outcomes such as lymph node status, treatment response, or recurrence risk. Unsupervised models can identify patient subgroups with shared imaging, exposure, immune, or molecular characteristics. Deep learning can learn features directly from raw images and integrate them with structured data (8, 44). Comparative evidence from medical imaging also suggests that deep learning systems should be judged against clinical standards and real-world performance requirements (4). Pathology-based deep learning studies further show that image-derived patterns can reflect spatial immune organization or molecular status (41, 45). When biological networks, exposure relationships, or cell-cell interactions are central to the research question, graph-based methods may also be useful (46) (**Figure 1**). The main applications, strengths, and limitations of these AI methods are summarized in **Table 1**.

**Figure 1:**
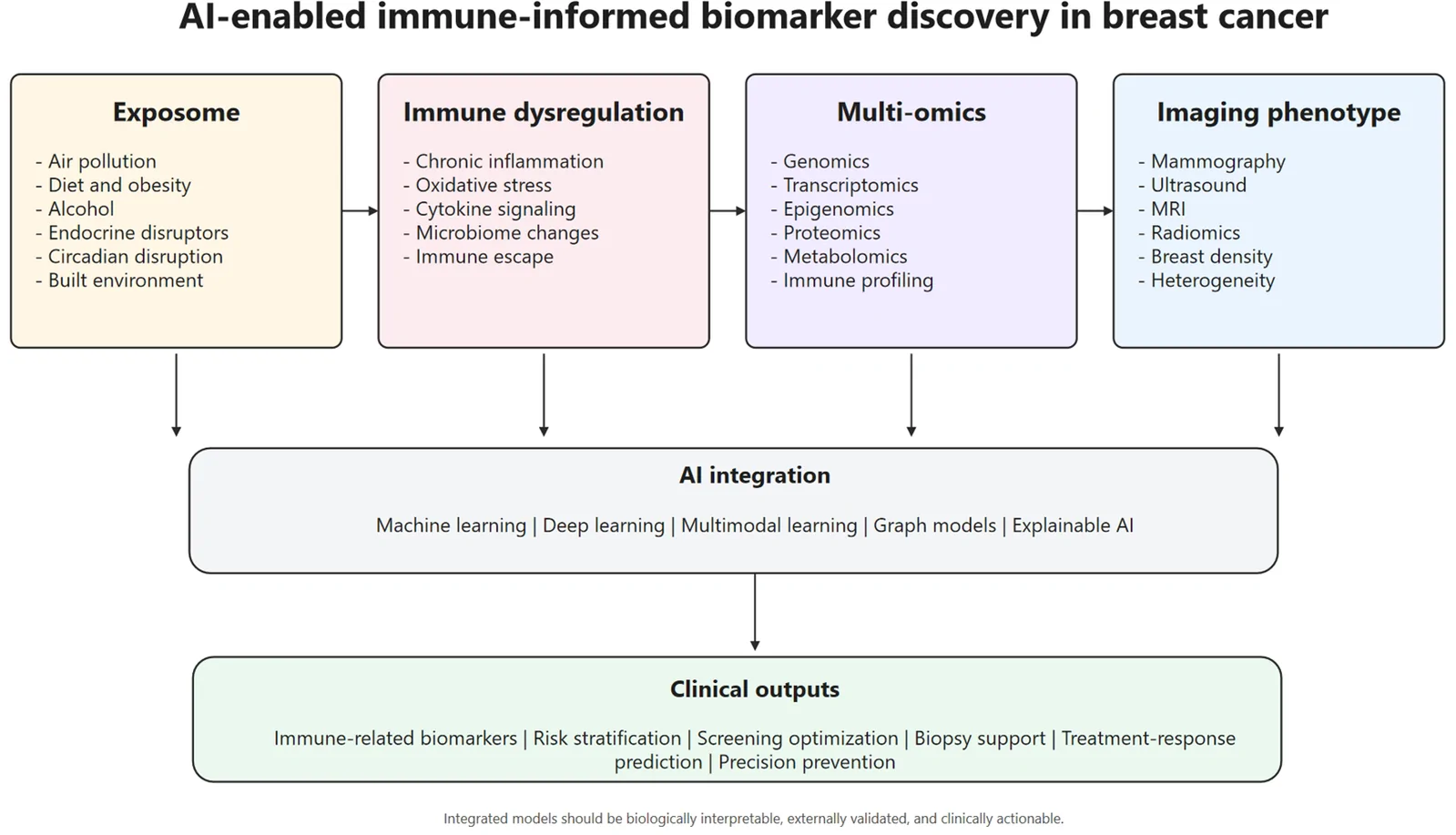
AI-enabled integration of imaging, exposome (environmental exposure profiling), and multi-omics data for the discovery of immune-mediated breast cancer biomarkers. Environmental and lifestyle exposures may affect inflammation, metabolism, microbiome composition, and immune regulation. Multi-omics data capture the molecular and immune consequences of these exposures, whereas imaging reflects downstream tissue-level phenotypes. AI can integrate information across these layers to support biomarker discovery, risk stratification, treatment response prediction, and precision prevention.

**Table 1:** Applications of AI methods for integrating imaging, exposome (environmental exposure profiling), and multi-omics data in breast cancer.

AI method	Integrated data types	Clinical applications	Strengths	Limitations
Supervised learning	Clinical variables, imaging features, selected molecular markers	Risk prediction, lymph node status assessment, treatment response prediction	Relatively easy to interpret clinically and can be aligned with existing endpoints	Depends on predefined endpoints and high-quality labeled data
Deep learning	Raw images combined with clinical or omics variables	Feature extraction and multimodal prediction	Can learn complex nonlinear patterns	Usually requires large datasets and rigorous external validation
Unsupervised learning	Imaging, exposure, immune, and molecular features	Patient subtype identification	Can reveal hidden phenotypes or new patient groupings	Clustering results may be unstable and difficult to interpret clinically
Graph-based models	Biological networks, exposure relationships, cell-cell interactions	Mechanism-based biomarker discovery	Can describe relationships among different entities	Model design and validation are complex
Explainable AI	Multimodal data	Clinical interpretation and hypothesis generation	Improves transparency and clinician trust	Explanations may be incomplete or misleading

For clinicians, model interpretability is critical. A model that only provides a high-risk result without explaining its basis is unlikely to be used confidently in patient counseling, biopsy decisions, or treatment planning. Explainable AI can help identify which imaging regions, exposure variables, molecular features, or immune pathways contribute more to the prediction (47). However, interpretation should not be limited to formal heatmaps or variable rankings. It should help determine whether the model captures tumor biology, patient-level risk, workflow differences, or dataset bias (23, 48).

Future models with greater value may not be those that only pursue higher predictive performance, but those that combine prediction with biological structure. For example, a model linking air pollution exposure, inflammatory transcriptomic profiles, breast density, and high-risk imaging phenotypes would have greater clinical meaning than an uninterpretable black-box score. Similarly, connecting radiomic heterogeneity with immune infiltration or cytokine pathways may provide clearer hypotheses for histological validation. These examples suggest that the next stage of research requires not only larger datasets, but also study designs that are closer to clinical questions.

## Challenges for translational medicine and future directions

Several practical barriers remain before integrated biomarkers can influence breast cancer diagnosis and treatment. Data fragmentation is probably the most immediate one. Imaging, pathology, sequencing, exposure records, treatment information, and outcome data are usually stored in different systems, with inconsistent formats and quality. This makes it difficult to build longitudinal datasets that reflect real clinical pathways. Generalizability is another concern. Breast imaging protocols, scanner vendors, reconstruction methods, patient populations, screening strategies, and treatment pathways differ across institutions. A model trained in one setting may lose performance when it is used in another center (49). This problem may be even greater for models that include exposome (environmental exposure profiling) information, because environmental exposures have clear geographic and social patterns. The evidence base also remains limited. Many AI studies are retrospective and rely mainly on internal validation. For clinical use, models need to show external validation, calibration, net benefit, and workflow feasibility (23). Reporting and appraisal frameworks such as TRIPOD+AI and PROBAST+AI emphasize transparent descriptions of data sources, model development, validation strategy, intended use, risk of bias, and applicability (48, 50). Fairness should also be considered from the beginning. Exposome and social environmental variables may improve risk prediction, but they may also reflect structural inequality. If a model performs poorly in underserved populations, it may widen existing gaps in screening, diagnosis, and prevention. Therefore, model performance, calibration, and clinical consequences should be assessed across different subgroups before implementation (51, 52).

Future research should place greater emphasis on longitudinal and clinically actionable models. Repeated imaging, serial molecular profiling, wearable device data, geospatial exposure assessment, and electronic health records may jointly support dynamic risk assessment. Causal inference methods may help distinguish exposure-related biomarkers from true pathogenic mechanisms (51). Spatial omics and single-cell technologies may also help explain how environmental exposures reshape immune and stromal ecosystems in breast tissue (53).

Prediction is not the same as explanation. While high-accuracy imaging models are useful, understanding underlying biology requires integrating imaging with multi-omics, exposome, and immune profiling. Additionally, study limitations and future research directions should be clearly outlined. For breast cancer, the practical goal is straightforward. AI should help clinicians identify high-risk patients earlier, understand tumor biology more clearly, choose appropriate imaging and biopsy strategies, reassess treatment response when needed, and support fair precision prevention. To reach this goal, models must do more than perform well in a dataset. They need biological interpretability, external validation, and a realistic place in the clinical workflow.
